# The Mental Health Impact of the COVID-19 Pandemic Across Different Cohorts

**DOI:** 10.1007/s11469-020-00367-0

**Published:** 2020-07-09

**Authors:** Kiran Shafiq Khan, Mohammed A. Mamun, Mark D. Griffiths, Irfan Ullah

**Affiliations:** 1 Dow Medical College, Dow University of Heath Sciences, Karachi, Pakistan; 2 Department of Public Health and Informatics, Jahangirnagar University, Savar, Dhaka Bangladesh; 3 Undergraduate Research Organization, Gerua Rd, Savar, Dhaka 1342 Bangladesh; 4 Psychology Department, Nottingham Trent University, 50 Shakespeare Street, Nottingham, NG1 4FQ UK; 5 Kabir Medical College, Gandhara University, Peshawar, Pakistan

The World Health Organization (WHO) defines mental health as the state of wellbeing in which an individual realizes their capabilities to combat with normal life stressors and work competencies in contributing to the belonged community, which is underpinned by six psychological elements comprising (i) self-acceptance, (ii) meaning in life, (iii) autonomy, (iv) healthy relationships with others, (v) environmental mastery, and (vi) personal growth (Mukhtar 2020). These mental health and emotional issues are now among the foremost public health concerns throughout the world because of the novel coronavirus 2019 (COVID-19) pandemic, due to fear of infection or fear of death from the virus. Consequently, many individuals are suffering from elevated anxiety, anger, confusion, and posttraumatic symptoms (Mukhtar 2020; Pakpour and Griffiths 2020). Studies have reported that the spatial distancing, self-isolation, quarantine, social and economic discord, and misinformation (particularly on social media) are among the major contributing factors towards unusual sadness, fear, frustration, feelings of helplessness, loneliness, and nervousness (Ahorsu et al. 2020; Sakib et al. 2020). In extreme cases, it may trigger suicidal thoughts and attempts and, in some cases, actually result in suicide (Bhuiyan et al. 2020; Dsouza et al. 2020; Griffiths and Mamun 2020; Mamun and Griffiths 2020; Mamun and Ullah 2020). In addition, the number of domestic violence cases has tripled during lockdown in China (Lee 2020), and that females are suffering great psychological strain (Ahorsu et al. 2020). Another Indian study claimed that females have a greater level of stress, anxiety, and depression compared to males. This was attributed to quarantine where all family members are at home which can be both mentally and physically demanding (Suseela 2020). However, the present paper briefly reviews the mental health problems faced across different cohorts and groups including (i) the general population; (ii) healthcare personnel; (iii) university and college students; (iv) schoolchildren; (v) hospitality, sport, and entertainment industry employees; and (vi) other vulnerable groups.

## General Population

Among the general population, COVID-19 transmission fear has facilitated the development of psychiatric symptoms such as depression, confusion, stress, and anxiety among individuals who have never previously experienced mental illness (Shigemura et al. 2020). At a community level, individuals may become suspicious towards others in terms of disease spread. Some individuals may develop fear of becoming very ill or dying helplessly. They may also blame one another and develop aggressive behavior towards people who are ill or perceived as ill (Ho et al. 2020), and this may also aggravate severe mental and emotional distress. The shutting of municipal services and the sudden collapse of industries have also contributed to changes in people’s mental health because a large number of individuals have experienced financial losses and unemployment that can further escalate emotional problems (Kawohl and Nordt 2020). Additionally, those confirmed as having COVID-19 may also undergo great psychological pressure and disturbance (Li et al. 2020). Other groups that may suffer psychologically are those who are anxiously awaiting their test results (Tang et al. 2018). Finally, the families of those who have suffered family deaths are also likely to experience immense grief and mental distress.

## Healthcare Personnel

Front-line healthcare employees such as doctors, nurses, first responders, paramedics, and ambulance personnel have also experienced excessive stress and have reported the highest level of depression and anxiety compared to low-risk healthcare workers working in chest and pulmonology departments (Pappa et al. 2020), alongside the anxiety and fear of getting infected with COVID-19 because of their increased risk of exposure (Ho et al. 2020). They fear becoming the source of infection to their loved ones especially children and family members who are older, immunocompromised, or chronically ill. Healthcare workers who work in intensive care units, emergency departments, and isolation wards are at greater risk of developing psychological breakdowns comprising severe fatigue, sleep disturbance, health concerns, and fear of contact with COVID-19 patients compared to those in other healthcare departments (Ho et al. 2020) because of the constant fear of getting an infection that leads to serious illness. Shortages of personal protective equipment intensify the fear, and there are significant shortages of masks, disinfectants, and other safety aids in many countries, making the workplace environment a more dangerous environment to work in (Cao et al. 2020).

Medical healthcare workers who are in direct contact with the positive COVID-19 cases are vulnerable to both physical infection and mental stress. In order to combat this, hospitals should provide physical spaces to all front-line healthcare workers where they can isolate after their work has finished for the day. This may reduce the disease transmission to some extent because healthcare workers are at high risk of becoming infected. For the sake of their mental health, counseling and psychotherapy based on stress adaptation techniques should be arranged by the hospital management to help address their psychological concerns and meet their mental health needs.

To deal with patients displaying antisocial behavior (e.g., verbal and physical abuse caused by the mental strain they may be feeling), healthcare facilitators require strict action to be taken by the hospital management. They should maintain a high-alert security force that ensures safety of all doctors, nurses, and all auxiliary staff on duty. Healthcare workers should have enough personal protective equipment to reduce fear among the healthcare employees.

## University and College Students

Among students, the strict isolation measures have meant that schools, colleges, and universities across the world have temporarily closed and this is predictably affecting their mental health (Cao et al. 2020). Students who are studying far away from their families suffer the most (Sahu 2020). The fear of getting infected and not seeing their families again for an unknown amount of time have affected them psychosocially (Zhai and Du 2020). They also have many concerns for the safety and health of their families (Zhai and Du 2020). A survey among 7143 medical college students (aged 19–25 years) reported that 24.9% experienced anxiety because of the COVID-19 outbreak (0.9% experiencing severe anxiety, 2.7% moderate anxiety, and 21.3% mild anxiety; Cao et al. 2020). For college and university students, postponement of events such as study exchanges and graduation ceremonies is also a factor that contributes to poor mood states. Some students have lost their part-time jobs because local businesses have closed (Lee 2020). The delivery mode from face-to-face classroom teaching to online classes has become an obstacle for some students especially for those living in remote areas that do not have access to laptops and Internet facilities at home. This can cause mental stress in terms of not being able to attend online classes (Sahu 2020).

## Schoolchildren

Although evidence suggests children and adolescents are less vulnerable to infection from COVID-19 (Ghosh et al. 2020), their psychosocial functioning has also been affected during the pandemic. Quarantine periods, school closures, lack of outdoor activities, aberrant diets, disrupted sleeping habits, domestic violence, and child abuse have resulted in monotony, distress, impatience, annoyance, and neuropsychiatric problems for some children and adolescents (Ghosh et al. 2020). Adolescents with mental health problems prior to the pandemic need special attention to help cope with quarantine because such closures mean a lack of access to the resources they had in school (Lee 2020). School routines distract such individuals from their illness, and not being able to attend school can result in stressful life situations and family conflicts. When schools are closed, their symptoms could relapse, and adolescents with depression may again lock themselves in their rooms and refuse to take showers or eat meals. Those dealing with autism spectrum disorder may face difficulties in adjusting back to normal life after the lockdown eases (Lee 2020).

## Hospitality, Sport, and Entertainment Industry Employees

Industry closures due to the pandemic (e.g., restaurants, bars, theaters, cinemas, gyms, shopping malls, and other public centers) have caused both temporary and permanent unemployment and great harm to national economies, especially developing countries (Gostin and Wiley 2020). Until a vaccine or effective treatment for COVID-19 is found, the hospitality and entertainment industries will have to revise their policies because spatial distancing will be the norm for the foreseeable future. The economic strain has had a significant impact on mental health (Jain 2020). Some sectors may suffer more than others (e.g., street food vendors because of unhygienic environment that is involved in preparing food) (Jain 2020). Regular health checkups and testing will become part of daily life, and cleanliness certification from food and drug authorities will become a mandatory part of the protocols for all food centers (Jain 2020). This new way of living will affect the psychological functioning of many individuals.

Government health authorities have banned all large-scale gatherings. Even when lockdowns are eased, spatial distancing is going to be part of modern life and the “new normal.” This affects religious worship, entertainment industry, sporting events, business meetings, and political rallies (Gostin and Wiley 2020). Again, the mental burden of not being able to meet up socially or watch live sport and entertainment is (at present) unknown. The entertainment and sporting industries have faced multiple drawbacks. Financial consequences have already been felt by sports clubs, studios owners, filmmakers, and theaters (Moon 2020). All sporting events, concerts, and public food festivals have been postponed and canceled until further notice (Moon 2020).

## Vulnerable Groups

Groups that are more vulnerable to getting severe COVID-19 symptoms include individuals that can be easily hurt physically or emotionally (Disu et al. 2019). Elderly people (aged over 65 years), homeless individuals, those with preexisting health conditions, and those living in care homes are included in this group. Just being in a vulnerable group may cause mental health problems (Tsai and Wilson 2020). Many homeless individuals already experience chronic mental and health conditions including bipolar disorder and schizophrenia. Compared to those in the general population, these individuals are more likely to engage in substance abuse and sharing of needles that make them more vulnerable towards infection including COVID-19 (Tsai and Wilson 2020). Individuals with chronic health conditions such as asthma, hypertension, diabetes, and chronic obstructive pulmonary disease are more susceptible to coronavirus infections (Tsai et al. 2019), and individuals with comorbidities have a high chance of getting infected than the general population (Guan et al. 2020). As yet, there is no way of knowing what the long-term mental health effects will be on these vulnerable groups but is likely to be significant.

## Final Thoughts

Media coverage of the pandemic can also influence the public’s mental health. The 24/7 coverage of COVID-19 on rolling news channels, sensationalist headlines in national newspapers, and misinformation on social media have also stimulated anxiety and fear among the general public (Ho et al. 2020). Websites hosting misinformation (e.g., false COVID-19, treatment remedies, COVID-19 conspiracy theories) should be removed and banned. Official media outlets should serve as an essential dissemination channel to encourage precautionary and preventive measures (Ho et al. 2020). Regular public engagement by government officials and scientific experts is needed to alleviate doubts (Ho et al. 2020). Inadequate knowledge concerning COVID-19 and rolling news may add anxiety and fear among the general public (Tang et al. 2018). Government health agencies should also make online counseling sessions available to help ease the mental health concerns and worries of the general population (Li et al. 2020).
